# Epigenetic regulation of thrombo-inflammation in Behçet and antiphospholipid syndrome

**DOI:** 10.1016/j.jtauto.2025.100293

**Published:** 2025-05-24

**Authors:** Alessandra Bettiol, Giacomo Bagni, Francesca Di Patti, Elena Lastraioli, Alice Barinotti, Massimo Radin, Savino Sciascia, Domenico Prisco, Annarosa Arcangeli, Giacomo Emmi

**Affiliations:** a Department of Experimental and Clinical Biomedical Sciences “Mario Serio”, University of Florence, Italy; b Department of Clinical and Biological Sciences, University of Turin, Turin, Italy; c Department of Mathematics and Informatics “U. Dini”, University of Florence, Italy; d National Institute for Nuclear Physics, Florence Division, Florence, Italy; e CSDC–Centro Interdipartimentale per lo Studio Delle Dinamiche Complesse, Florence, Italy; f Department of Experimental and Clinical Medicine, University of Florence, Italy; g Center of Research of Immunopathology and Rare Diseases, Department of Clinical and Biological Sciences, and SCDU Nephrology and Dialysis, S. Giovanni Bosco Hospital, Turin, Italy; h University of Turin, Torino, Italy; i Internal Interdisciplinary Medicine Unit, Behçet Centre, Careggi University Hospital, Florence, Italy; j Department of Medical, Surgery and Health Sciences, University of Trieste, Italy; k Clinical Medicine and Rheumatology Unit, Cattinara University Hospital, Trieste, Italy; l Centre for Inflammatory Diseases, Monash University Department of Medicine, Monash Medical Centre, Melbourne, Australia

**Keywords:** Behçet syndrome, Antiphospholipid syndromes, Epigenetics, miRNA, Thrombosis

## Abstract

**Background:**

An epigenetic regulation of thrombo-inflammation has been reported in Behçet syndrome (BS), likely driven by a unique profile of three plasmatic circulating microRNAs (ci-miRNAs) (miR-206, miR-224-5p, and miR-653-5p). We compared this ci-miRNAs expression in BS and antiphospholipid syndrome (APS), the prototype of acquired pro-thrombotic autoimmune disease. To further corroborate the hypothesis that shared mechanisms drive the thrombotic process in BS and APS, we further assessed their thrombin generation assay (TGA) profile.

**Methods:**

The three ci-miRNA expression was evaluated in 39 patients with BS, 33 with APS and 30 healthy controls (HCs). Single marker and combined ROC curve analyses were performed. TGA was conducted on pre-collected platelet poor plasma samples from 35 patients with BS and 77 with APS.

**Results:**

The three ci-miRNAs, taken individually or combined, lacked acceptable discriminating power between groups [AUC from combiROC 0.64 (95 % CI: 0.51–0.78)]. Conversely, in the subgroups of BS and APS patients with vascular involvement (n = 22 each), the combined signature well discriminated between the two syndromes [AUC 0.83 (0.71–0.96), specificity 0.91, sensitivity 0.77], as well as between thrombotic APS and HCs [AUC 0.79 (0.64–0.91)]. Also, distinct trends in thrombograms emerged between BS and APS, with BS TGA displaying lower tLag and tPeak, higher Peak and similar AUC as compared to APS.

**Conclusions:**

Despite shared elements in the ci-miRNA regulation of their pro-thrombotic tendency, distinct epigenetic factors seem to contribute to the pathogenesis of vascular events in BS and APS, possibly accounting also for the different global clotting assay observed in these diseases.

## Highlights

• Three ci-miRNAs are known to regulate thrombo-inflammation in Behçet syndrome (BS).
• This ci-miRNA signature is also present in antiphospholipid syndrome (APS).
• These ci-miRNA signature discriminates BS with vascular events from thrombotic APS.
• Distinct curves in global clotting assay (thrombogram profile) emerge in BS and APS.
• Distinct epigenetic factors seem to regulate thrombosis in patients with BS and APS.

## Glossary

BS: Behçet syndrome
ROS: reactive oxygen species
NETs: neutrophil extracellular traps
ci-miRNAs: circulating microRNAs
SLE: systemic lupus erythematosus
APS: antiphospholipid syndrome
GCA: giant cell arteritis
ICBD: International Criteria for Behçet Disease
HC: healthy control
PPP: platelet poor plasma
BDCAF: Behçet Disease Current Activity Form
aPL: antiphospholipid antibodies
HCQ: hydroxychloroquine
DMARDs: disease-modifying antirheumatic drugs
PFP: Platelet-free plasma
IQR: interquartile range
tLag: Lag time
tPeak: time to peak
AUC: area under the curve
TGA: Thrombin generation assay
combiROC: combined Receiver Operating Characteristic

## 1 Introduction

Behçet syndrome (BS) is a rare chronic inflammatory disease of unknown aetiopathogenesis, classified among systemic vasculitides. The first described triad of BS-related manifestations include oral and genital ulcers and uveitis, although BS can virtually affect any organ [[1], [2], [3]]; among others, vascular manifestations can occur in up to 50 % of patients, mostly in the form of superficial and deep vein thrombosis [4].

BS is currently considered as a model of thrombo-inflammation, as thrombogenesis seems to be mostly mediated by an impaired immune-inflammatory response rather than traditional cardiovascular risk factors [4,5]. In particular, neutrophil hyperactivation and neutrophil-mediated damage, including an enhanced production of reactive oxygen species (ROS) [6,7] and neutrophil extracellular traps (NETs) [8], sustain this process. Additionally, ROS can oxidate fibrinogen, altering its structural and functional features in a pro-thrombotic sense [6].

An epigenetic regulation via circulating microRNAs (ci-miRNAs) has been recently suggested in several autoimmune diseases [[9], [10], [11], [12], [13], [14], [15], [16], [17], [18]]. We described a unique profile of three plasmatic ci-miRNAs (namely miR-206, miR-224-5p, and miR-653-5p) acting as post-transcriptional regulators of gene expression in molecular pathways related to cell-matrix interaction, oxidative stress and blood coagulation [19]. Moreover, a direct correlation between the expression levels of these ci-miRNAs and both leucocyte ROS production and plasma lipids peroxidation was reported [19,20], further strengthening the hypothesis that these ci-miRNAs regulate thrombo-inflammatory mechanisms associated with vascular BS. We also showed that this ci-miRNA signature is able to discriminate BS patients from systemic lupus erythematosus (SLE) and giant cell arteritis (GCA) patients [19]. This suggests that such ci-miRNA profile regulates pathogenetic mechanisms involved in BS, which are not shared with other representative systemic autoimmune diseases or vasculitides.

Given the natural tendency to promote thrombosis in BS, we assessed the expression of the above ci-miRNA profile in patients with antiphospholipid syndrome (APS), the prototype of acquired pro-thrombotic autoimmune disease [21,22]. APS has been shown to share a common miRNA signature with thromboembolic diseases, with some miRNAs (miRs-124-3p, 125b-5p, 125a-5p, 126a-3p, and 17-5p) being associated with APS and arterial thrombosis, and some others (miRs-106a-5p, 126a-3p, 146b-5p, 15a-5p, 222-3p, and 451a) with venous thrombosis [23].

In this study, we wanted to explore whether the mi-RNA signature identified in BS is present or not in APS, with the underlying hypothesis that a different ci-miRNA expression might indicate distinct pathogenetic mechanisms sustaining the thrombotic tendency in these two conditions. To further corroborate the hypothesis that different mechanisms drive the thrombotic process in BS and APS, a sub-analysis was performed assessing the thrombin generation curve (i.e thrombogram) profile in the two diseases.

## 2 Methods

### 2.1 Study design and population

A cross-sectional study aimed at assessing the epigenetic profile of BS *vs* APS patients was conducted on a cohort of 39 patients with BS fulfilling the International Criteria for Behçet Disease (ICBD) [24] who were referred to the Behçet Centre of the Careggi University Hospital (Florence, Italy). Thirty-three patients with APS meeting the revised Sapporo criteria for APS [21], and 30 healthy controls (HCs) were also included. Subjects with other systemic vasculitis/autoimmune diseases, active infections or neoplastic conditions were excluded.

In a further cross-sectional sub-study, previously collected platelet poor plasma (PPP) samples from a group of 35 BS patients and 77 APS patients were analysed to assess and compare the thrombin generation profile in these two diseases.

The work was conducted in accordance with the Code of Ethics of the World Medical Association (Declaration of Helsinki) for experiments involving humans and received approval by the local ethics committee (ref n. 13972). All subjects included in both sub-studies provided written informed consent before data and sample collection.

### 2.2 Clinical assessment

At the time of sample collection, all patients underwent a review of their demographic and medical data, including a comprehensive assessment of clinical involvement in the whole medical history and of active disease manifestations at the time of enrolment. Specifically, for BS patients, the presence of mucosal, cutaneous, articular, gastrointestinal, ocular, neurological and vascular (venous or arterial) involvement in the whole medical history was recorded. Also, the HLA-class I status was investigated. Disease activity at the time of enrolment was assessed based on the Behçet Disease Current Activity Form (BDCAF), with active disease defined as BDCAF score ≥1 [25]. For APS patients, the history of vascular events and/or pregnancy complications along with information on antiphospholipid antibodies (aPL) positivity (single, double, or triple positivity) was collected. Moreover, pharmacological history of ongoing therapies at the time of inclusion in the study was recorded for both syndromes, with a particular focus on corticosteroids, hydroxychloroquine (HCQ), colchicine, traditional or biological disease-modifying antirheumatic drugs (DMARDs), anticoagulants and antiplatelet agents.

### 2.3 Sample collection and ci-miRNA analyses

All BS and APS patients and HCs underwent peripheral blood sample collection (8 mL) in K2-EDTA anticoagulant by standard venipuncture. Blood samples were processed within 2 h from collection. Platelet-free plasma (PFP) was obtained by double-step centrifugation protocol (1500 g for 15 min at room temperature followed by supernatant centrifugation at 13 000 g for 3 min), and total RNA was extracted from 250 μl of plasma using TRIzol LS reagent (Invitrogen, Carlsbad, California, USA) following the manufacturer's indications, as previously described [19]. Only samples with acceptable RNA quality and concentration were finally included in the study. Plasma samples showing signs of hemolysis were excluded from analysis.

The expression levels of circulating miR-206, miR-224-5p, and miR-653-5p were quantified by Poly(T) Adaptor RT-qPCR using the retro-transcription protocol, mature miRNA-specific primers design and thermal Real-Time PCR protocol as previously described [19].

### 2.4 Thrombin generation assay (TGA)

TGA was performed following the procedures reported in a previous study [26] using a commercially available assay kit (Technothrombin TGA kit, Techonoclone, Vienna, Austria). The assay allows dynamic simultaneous recording of the effects of both thrombin formation and inhibition, thus reflecting the *in vivo* result of pro‐ and anticoagulant activities and the consequent haemostasis status of a patient [27]. Thanks to these unique properties, TGA has been applied for diagnostic, prognostic and -monitoring purposes in various inherited and acquired coagulopathies [28], including APS, often coupled with aPL assays to provide a more sensitive and informative diagnostic work up [[29], [30], [31], [32], [33]].

Indeed, traditional laboratory markers such as the prothrombin time (PT) and the activated partial thrombopastin time (aPTT) present limitations in assessing the thrombotic risk in conditions such as APS, where the presence of a lupus anticoagulant results in aPTT prolongation but paradoxically determines an increased thrombotic (and not bleeding) risk [28]. Thus, TGA is considered a highly representative model of *in vivo* physiology of coagulation in conditions such as APS, although lack of standardization of pre-, analytical, and post-analytical factors is sometimes reported as a possible limitation [28].

In this work, TGA was performed on a fully automated, computer-controlled microplate reader with dedicated software (Technothrombin TGA, Vienna, Austria) and utilized a fluorogenic substrate Z-Gly-Gly-Arg-AMC (Bachem, Bubendorf, Switzerland). In the Technoclone assay, 40 μL of PPP was combined with 10 μL of activation reagent (RB Low reagent), followed by the addition of 50 μL of calcium–fluorescent substrate reagent (resulting in final reaction concentrations of 7.5 mmol/L calcium and 0.5 mmol/L substrate) to initiate the reaction. Thrombin concentration was recorded over time, generating a thrombin generation curve, from which several parameters could be estimated, including: i) Lag time (tLag), defined as the time of the delay phase following the addiction of the trigger until the start of thrombin generation; ii) Time to Peak (tPeak), the time to reach the peak, representing the velocity of thrombin generation; iii) Peak, representing the highest thrombin concentration generated; iv) Area under the curve (AUC), the total amount of thrombin generated.

### 2.5 Statistical analysis

Categorical variables were presented as absolute frequencies and percentages. Continuous variables were reported as median value and interquartile range (IQR) and were compared between the three groups using the Kruskal-Wallis test for unpaired data, followed by head-to-head post-hoc Dunn's multiple comparison test. Data distribution was checked using the Shapiro-Wilk test.

ROC curve analysis was performed using Matlab built-in function perfcurve V.2019a. Statistical analyses were performed using the Graph Pad Prism V.6.0 (GraphPad Software, San Diego, California, USA) and the software STATA version 14. p-values and adjusted p-values <0.05 (in overall analysis) were considered for statistical significance.

## 3 Results

### 3.1 Study population

The epigenetic study included 39 patients with BS, 33 with APS and 30 HCs, whereas the TGA analysis was conducted on a pre-collected cohort of 35 patients with BS and 77 with APS. Clinical and demographic characteristics of patients included in the two analyses are reported in Table 1. Regarding patients included in the epigenetic study (i.e ci-miRNA analysis), sex distribution and median age at inclusion in the study were comparable between BS and APS patients and HCs (female representing 56.4–57.6 % of included patients), with a median disease duration of 6 (IQR 3–10) and 4 (IQR 1–15) years in the BS and APS cohorts, respectively. Conversely, the great majority of patients included in the TGA analysis were female (88.6 % and 89.6 % with BS and APS, respectively), with a median disease duration of 5 (IQR 2–12) and 6 (IQR 1–12) years, respectively.

**Table 1:** Demographic, clinical and therapeutic characteristics of patients included in the ci-miRNAs and in the TGA analyses.

	Included in the ci-miRNAs analysis			Included in the TGA analysis	
	BS patients (n = 39)	APS patients (n = 33)	Healthy controls (n = 30)	BS patients (n = 35)	APS (n = 77)
Female sex, n (%)	22 (56.4 %)	19 (57.6 %)	17 (56.7 %)	31 (88.6 %)	69 (89.6 %)
Age at inclusion, median (IQR)	43 (34–51)	38 (33–52)	44 (37–52)	46 (28–55)	41 (31–55)
Disease duration, years median (IQR)	6 (3–10)	4 (1–15)		5 (2–12)	6 (1–12)

**Vascular events**, n (%)	22 (56.4 %)	22 (66.7 %)		19 (54.3 %)	77 (100 %)
Venous	18 (46.2 %)	19 (57.6 %)		15 (42.9 %)	55 (71.4 %)
Arterial	6 (15.4 %)	7 (21.2 %)		4 (11.4 %)	22 (28.6 %)
**Obstetric APS**, n (%)	–	11 (33.3 %)		–	10 (14.5 %)

**Other disease manifestations in BS patients**, n (%)					
Mucosal	37 (94.9 %)	–		35 (100 %)	–
Cutaneous	26 (66.7 %)	–		31 (88.6 %)	–
Articular	19 (48.7 %)	–		22 (62.9 %)	–
Ocular	15 (38.5 %)	–		17 (48.6 %)	–
Neurological	12 (30.8 %)	–		7 (20.0 %)	–
Gastrointestinal	6 (15.4 %)			3 (8.6 %)	
**HLA-B51**, n (%)	17 (43.6 %)	–		15 (42.9 %)	–

**Antiphospholipid antibodies**, n (%)					
Single	–	6 (18.2 %)		–	1 (1.3 %)
Double	–	9 (27.3 %)		–	21 (27.3 %)
Triple	–	18 (54.5 %)		–	55 (71.4 %)

**Active disease at enrollment**, n (%)	19 (48.7 %)	–		15 (42.9 %)	–

**Ongoing pharmacological therapies**, n (%)					
Corticosteroids	19 (48.7 %)	–		19 (54.3 %)	–
HCQ	–	12 (36.4 %)		–	7 (9.1 %)
Colchicine	8 (20.5 %)	–		9 (25.7 %)	–
Traditional DMARDs	17 (43.6 %)	–		18 (51.4 %)	–
Biological DMARDs	30 (76.9 %)	–		26 (74.3 %)	–
Anticoagulants	3 (7.7 %)	17 (51.5 %)		2 (5.7 %)	77 (100 %)
Antiplatelets	1 (2.6 %)	5 (15.2 %)		3 (8.6 %)	22 (28.6 %)

Regarding patients included in the epigenetic study, vascular involvement within the entire medical history was reported in 22/39 (56.4 %) patients with BS and in 22/33 (66.7 %) patients with APS. Specifically, venous events occurred in 18 (46.2 %) patients with BS and in 19 (57.6 %) with APS, while arterial events were found in six (15.4 %) patients with BS and seven (21.2 %) with APS, respectively. The remaining 11 (33.3 %) APS patients had a history of only obstetric complications.

As for patients included in the TGA analysis, vascular events had occurred in 19/35 (54.3 %) patients with BS and in all the 77 patients with APS, most events being venous (15/19 and 55/77, respectively); 10 patients with APS had a concomitant history of thrombotic and obstetric complications.

Within the cohorts of BS patients included in the epigenetic and in the TGA analyses, other common disease manifestations included mucosal lesions (94.9–100 %), followed by cutaneous (66.7–88.6 %%), articular (48.7–62.9 %), ocular (38.5–48.6 %) and neurological (30.8–20.0 %) involvement.

At the time of inclusion in the study, almost half of the patients with BS included in the epigenetic and in the TGA analyses, respectively, had active disease (19/39, 48.7 %, and 15/35, 42.9 %). Most of them were receiving biological and/or traditional DMARDs, and 4/39 and 5/35 BS patients included in the epigenetic and in the TGA analyses were on anticoagulant or antiplatelet therapy, respectively. Conversely, in the APS cohort, most patients were treated with anticoagulants (51.5 %–100 %) and 15.2 %–28.6 % of patients were receiving antiplatelet therapy.

### 3.2 Expression analysis of the three ci-miRNAs

The expression levels of miR-206, 224-5p and 653-5p were assessed and compared in patients with BS, APS and in HCs. The three cohorts displayed a significant different distribution in the expression levels of the miR-206 and 224-5p, but not of the miR-653-5p (p values from Kruskal Wallis test of 0.004, 0.006 and 0.067, respectively) (Fig. S1). In the head-to-head post-hoc Mann-Whitney analyses, miR-206 was found to be significantly deregulated in patients with APS as compared to HCs, while miR-224-5p significantly differed in BS and HCs; conversely, no significant difference was found between BS and APS when the three ci-miRNAs were analysed as single markers.

**Fig. 1:**
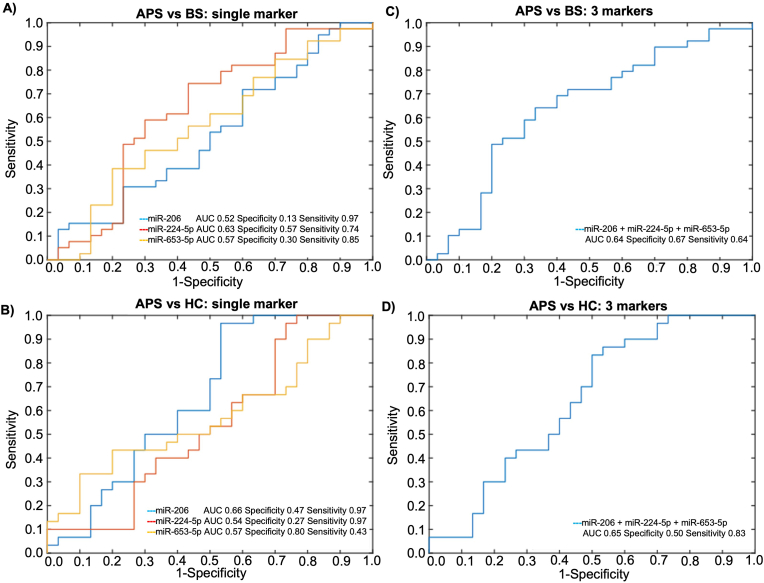
Single (**A and B**) and combined (**C and D**) ci-miRNAs ROC curve analysis showing the capability of miR-206, miR-224-5p and miR-653-5p to discriminate between BS patients (n = 39), APS patients (n = 33) and healthy controls (n = 30).

Coherently, when assessing the accuracy of these ci-miRNAs, taken individually, in discriminating BS and APS patients, we found very low AUC values for all the three mi-RNAs [for miR-206: 0.52 (95 % CI: 0.39–0.66); for miR-224-5p: 0.63 (95 % CI: 0.50–0.77); for miR-653-5p: 0.57 (95 % CI:0.43–0.70)], with extremely low specificity values (0.13, 0.57 and 0.30 for the three mi-RNAs, respectively) (Fig. 1A). Similarly, the single ci-miRNAs failed to reach a good discriminatory power between APS and HC [AUC for miR-206: 0.66 (95 % CI: 0.52–0.79); for miR-224-5p: 0.54 (95 % CI: 0.39–0.68); for miR-653-5p: 0.57 (95 % CI:0.43–0.72); specificity of 0.47, 0.27 and 0.80, respectively; sensitivity of 0.97, 0.97 and 0.43)] (Fig. 1B).

### 3.3 Combined analysis of the three ci-miRNAs signature

We further investigated whether the signature defined by the combination of the three ci-miRNAs was able to discriminate BS from APS. In the combined ROC (combiROC) curve analysis, the three mi-RNAs signature displayed poor capability to discriminate BS from APS patients, the AUC from the combiROC being 0.64 (95 % CI: 0.51–0.78), with a fair specificity (0.67) and sensitivity (0.64) (Fig. 1C).

Concomitantly, the combination of the three selected mi-RNAs failed to successfully discriminate APS patients from HCs [AUC 0.65 (95 % CI: 0.51–0.79)] with a poor specificity (specificity 0.50) (Fig. 1D). Conversely, the three mi-RNAs successfully discriminated BS patients from HCs, as already reported in our previous work [9] (*data not shown*).

### 3.4 Subgroup analysis of the ci-miRNA signature in patients with vascular involvement

To further investigate the hypothesis that the absence of statistically significant differences of three ci-miRNA signatures between BS and APS might be related to a common tendency to thrombogenesis, a subgroup analysis was conducted only including BS and APS patients with history of vascular events (22 patients in each group, respectively) as compared to HCs.

No significant difference was found in the expression levels of the three ci-miRNAs, taken individually, between BS and APS patients with vascular involvement (Fig. S2). When comparing the expression levels to the HC group, ci-miR-206 was differentially expressed in both BS and APS patients as compared to HCs, and miR-653-5p in APS patients (but not in those with BS) as compared to HCs. As for miR-224-5p, a significantly different expression was detected between BS (but not APS) and HCs.

**Fig. 2:**
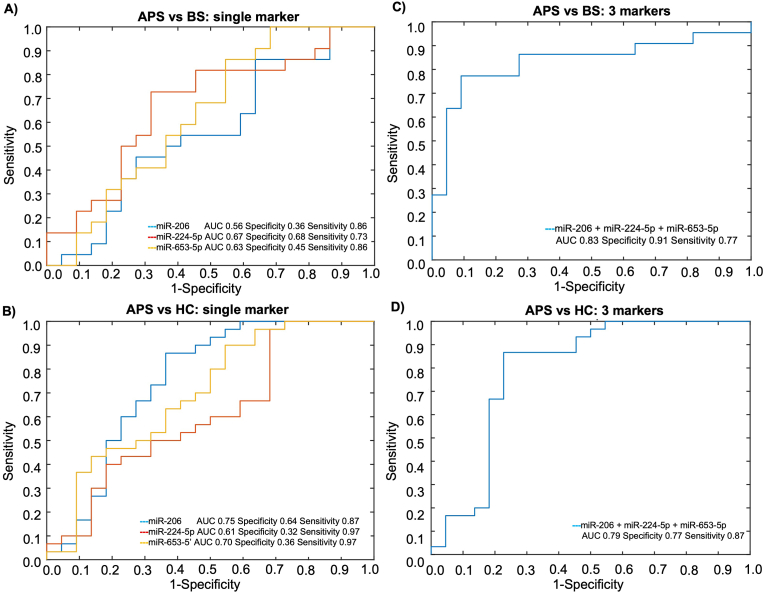
Single (**A and B**) and combined (**C and D**) ci-miRNAs ROC curve analysis showing the capability of miR-206, miR-224-5p and miR-653-5p to discriminate between the subgroups of BS (n = 22) and APS patients (n = 22) with vascular events and healthy controls (n = 30).

Coherently, when assessing the accuracy of these ci-miRNAs, taken individually, in discriminated BS and APS patients with history of vascular involvement, we found low AUC values for all the three mi-RNAs [for miR-206: 0.56 (95 % CI: 0.39–0.73); for miR-224-5p: 0.67 (95 % CI: 0.51–0.83); for miR-653-5p: 0.63 (95 % CI:0.47–0.80)], with very low specificity (0.36, 0.68 and 0.45 for the three mi-RNAs, respectively) (Fig. 2A).

Similarly, the single ci-miRNAs had a modest discriminatory power between APS and HC [AUC for miR-206: 0.75 (95 % CI: 0.61–0.89); for miR-224-5p: 0.61 (95 % CI: 0.45–0.77); for miR-653-5p: 0.70 (95 % CI: 0.55–0.85); specificity of 0.64, 0.32 and 0.36, respectively] (Fig. 2B).

Conversely, when combining the three ci-miRNAs in the combiROC curve analysis, the signature displayed a good capability to discriminate BS from APS patients, the AUC from the combiROC being 0.83 (95 % CI: 0.71–0.96), with a good specificity (0.91) and sensitivity (0.77) (Fig. 2C). Concomitantly, the combination of the three selected mi-RNAs discriminated APS patients from HCs with an AUC 0.79 (95 % CI: 0.64–0.91), a specificity of 0.77 and a specificity 0.87 (Fig. 2D).

We then focused on potential differences in the expression levels of the three ci-miRNAs within the BS and APS cohorts of patients with vascular involvement, sub-stratifying them according to the therapeutic features at the time of enrollment, as they remarkably differed between APS and BS patients.

Within the BS cohort, ci-miR-206 was differentially expressed in patients treated *vs* not treated with biological DMARDs (p = 0.021), while miR-224-5p and miR-653-5p tended to be differentially expressed in patients with *vs* without corticosteroid therapy (p = 0.041 and p = 0.048, respectively). No significant difference in the expression level of the three single mi-RNAs was found within the APS cohort, when stratifying patients according to the ongoing treatment with HCQ, anticoagulants or antiplatelet drugs (Table S1).

### 3.5 TGA analysis

**Fig. 3:**
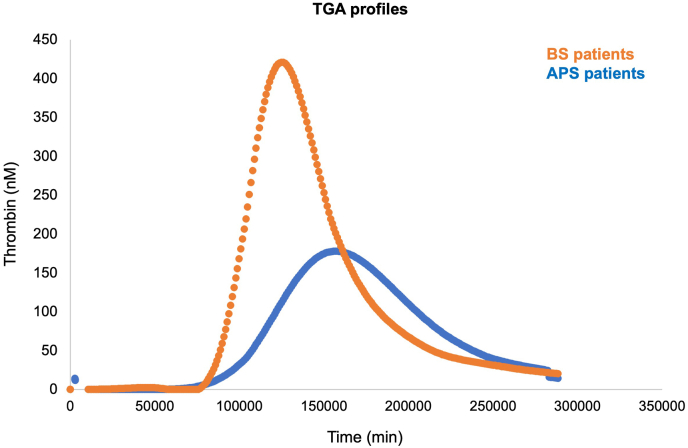
Thrombogram profile of BS and APS patients.

In a further sub-study, the thrombogram profile of BS and APS patients was compared (Fig. 3).

Two distinctive TGA profiled were observed, with lower tLag (tLag 2 ± 3.1 vs 9.7 ± 7.1 in BS and APS, respectively; p < 0.05) and tPeak (21.7 ± 7.1 vs 36.1 ± 15 in BS and APS, respectively; p < 0,05) and higher Peak (269.3 ± 167.5 vs 131.9 ± 161.0 in BD and APS, respectively; p < 0.05) in BS as compared to APS patients. Conversely, no significant changes were observed when comparing the AUC between the two disease groups (2139.8 ± 1234.8 vs 2317.5 ± 1071.4).

To exclude the potential confounding effect of ongoing anticoagulant therapy on TGA, a sub-analysis was conducted including only 12 cases with APS analysed before initiating anticoagulants, matched by age and sex with 12 BS patients not on anticoagulants (Table S2). The sub-analysis confirmed a trend toward higher peak values and shorter tLag in BS compared to APS.

## 4 Discussion

BS is known to be multifactorial disease, and various studies have shown that a dysregulation of ci-miRNAs expression might be a relevant mechanism in the multifaceted pathogenesis of this disease [34], 35. A downregulation of different miRNAs, including miR-155, miR-23b, and miR-196, paired by an upregulation of others, such as miR-21 and miR-181b, have been described [34].

In a previous study by our group, we confirmed a deregulated ci-miRNA profile in BS, and identified a new signature of three ci-miRNAs (miR-206, miR-224-5p and miR-653-5p) that segregated BS patients from HCs [19]. As these ci-miRNAs regulate the translation of proteins involved in cell-matrix interaction, oxidative stress and blood coagulation, a possible involvement of these epigenetic modulators in the thrombo-inflammatory mechanisms sustaining vascular BS was suggested. Moreover, the three ci-miRNAs positively correlated with leucocyte ROS and plasma lipid peroxidation, further supporting the association between this profile and thrombo-inflammation in BS [19].

In the present study, we showed that this ci-miRNA signature is present not only in BS, but also in APS patients. APS is the prototype of a pro-thrombotic autoimmune condition, with thrombi potentially affecting vascular beds of all sizes including arterial, venous, and microvascular vessels [21,22], similarly to BS. The pathogenesis of thrombotic events in APS seems to be sustained by a “two-hit” aetiology, in which antiphospholipid antibodies (aPL) represent the first hit as they create a generalized procoagulant state, and a second hit mediates the final thrombus formation [33]. Growing evidence supports the role of inflammatory stimuli as “second hit” factors in APS-related thrombogenesis, with multiple mediators giving a possible contribution: indeed, increased levels of endothelium-derived microparticles, platelet-leucocyte aggregates, tissue factor-expressing monocytes, complement split products as well as neutrophil products have been described in patients with APS [33],[36], [37], [38], [39], [47], [46], [45], [44], [43], [42], [41], [40]]. Among the latter, an enhanced release of NETs has been reported in APS, mostly mediated by aPL [36,37].

Neutrophils are well known key mediators of thrombo-inflammation in BS, and neutrophil-mediated release of ROS and NETs has been linked to structural and functional oxidation to fibrinogen, in a pro-thrombotic sense [19,20,38]. The results of the present study suggest that BS and APS share a common tendency towards thrombosis, possibly mediated by epigenetic mechanisms controlling the main thrombo-inflammatory pathways already identified in both conditions, namely, cell-matrix interaction, oxidative stress, and blood coagulation [[39], [44], [45], [46], [47]].

Accordingly, literature data support the notion that, in APS, a ci-miRNAs signature is present [43] and acts as regulator of a number of genes involved in key pathogenetic mechanisms of APS, such as immune response, atherosclerosis and thrombosis [42].

However, when considering only the subgroup of BS and APS patients who had already experienced vascular events, the three ci-miRNA signature showed a good capability to discriminate between the two syndromes. These findings might indicate that distinct factors intervene in modulating the epigenetic regulation of thrombosis in the two diseases. Among them, aPL might act as modulator of ci-miRNAs expression in APS. Indeed, ci-miRNAs have been shown to correlate with aPL titres, and to be modulated at least *in vitro* by aPL of IgG isotype [42]. Conversely, this modulatory interaction could not occur in BS, which lacks specific autoantibodies.

To complement our findings, the thrombogram profile was assessed in a cohort of BS patients (half of whom had history of vascular events) and in a cohort of patients with thrombotic APS. Our results clearly indicated distinct trends in thrombograms when comparing these two populations. The TGA profile in the APS cohort was consistent with previous findings in this condition [26,44], showing prolonged kinetic parameters (tLag and tPeak). A prolonged tLag indicates a delayed initiation of coagulation, while a prolonged tPeak indicates a greater time needed to reach the maximum thrombin concentration, suggesting slower starting of thrombin generation, amplification and propagation. These findings are likely related to activated protein C (APC) resistance due to the presence of lupus anticoagulant, as it interferes with phospholipid-dependent coagulation, inhibiting the formation of the prothrombinase complex, a key step in thrombin generation [44]. Accordingly, literature data indicate that aPL-positive subjects present a prolongation of tLag and tPeak but increased AUC, Peak, and velocity index as compared to health control, resulting in an overall procoagulant profile [41,40]. Notably, these kinetic parameters have been correlated with venous thromboembolism in the overall population [48,49], but are not sufficient to assess the thrombotic risk in aPL-positive subjects, further supporting the concept that distinct mechanisms are responsible for the final occurrence of thrombotic events in this disease [50]. Indeed, in some patients considered at high thrombotic risk (such as in those with triple positivity), a paradoxically decreased AUC and Peak have been reported, likely explained by decreased APC sensitivity [51], which is primary impaired by aPL via numerous mechanisms [40].

Conversely, the TGA profile in BS was characterized by low tLag and tPeak, and higher Peak as compared to APS. This profile indicates that the coagulation system is more easily activated and generates more thrombin than normal, and can be found with conditions marked by concurrent hypercoagulability, endothelial dysfunction, and inflammation [[52], [53], [54]]. Conversely, no differences in the AUC were observed between the two diseases.

A single study in literature assessed thrombin generation in 56 BS patients and 56 matched healthy controls, indicating a higher endogenous thrombin potential (i.e., AUC) in BS patients as compared to controls, and in BS patients with a history of thrombosis as compared to those without thrombosis [55]; however, no information on the other TGA kinetic parameters was reported.

Taken together, the results of these analyses confirm a pro-thrombotic status in both diseases, potentially regulated by common molecular pathways. Nevertheless, distinct epigenetic factors could contribute to the pathogenetic mechanisms leading to vascular events in BS and APS, possibly accounting also for the different findings in thrombogram between the two diseases.

This hypothesis needs to be further investigated; however, the differences in the current therapeutic approach to the cardiovascular prevention in the two conditions seems to support this notion. Indeed, in APS, anticoagulants represent the standard treatment of thrombosis [56]; on the other hand, in BS immunomodulation represents the mainstay for treating vascular manifestations, and the role of anticoagulation is widely debated [1,4,57].

Some limitations should be considered when interpreting these results. First, our study includes relatively small cohorts and investigates only three ci-miRNAs identified in a previous study by our group. The overall miRNome profile of APS was not investigated, and a future direct comprehensive comparison of ci-miRNA profile in APS *vs*. BS remains to be done in *ad hoc* studies. Second, no validation analysis was conducted in the APS cohort, and external validation of this signature in a larger independent cohort is required. Third, given the cross-sectional study design, the influence of natural variation of these ci-miRNA levels could not be assessed. Fourth, epigenetic and TGA analyses were conducted on distinct BS and APS cohorts, recruited in different clinical settings and calendar periods. Fifth, it is not possible to clearly identify the contribute of the different immunosuppressive, anticoagulant and antiplatelet therapies administered for the two conditions to the present results; indeed, it was not feasible to enrol only treatment-naïve patients, nor to stratify analyses for the different therapeutic subgroups due to the small sample size. Future studies on treatment-naïve cohorts of age-, sex- and comorbidities-matched BS and APS patients are advocated to confirm the present results. Sixth, the relatively small sample sizes of the BS and APS cohorts included in the TGA study prevented us from performing sub-group analyses, to assess the association between TGA kinetics and clinical manifestations and disease activity in the two diseases. Finally, a functional *in vitro* study is required to further strength the present results.

Despite these limitations, our results suggest for the first time that a three ci-miRNA profile might dictate the natural tendency to thrombotic events both in BS and APS, while distinct epigenetic factors could contribute to the pathogenesis of vascular events. These results might open the scenario for new studies assessing the role of these ci-miRNAs as novel biomarkers for monitoring the thrombotic risk in these conditions. Concomitantly, these ci-miRNAs might represent candidate therapeutic targets for thromboprophylaxis in these diseases. Previous literature data indicate that treatment with ubiquinol (the reduced form of coenzyme Q10, with antioxidant and anti-inflammatory properties) can modify the expression levels of some miRNAs having as potential target molecules involved in inflammation and thrombotic processes, suggesting that increased levels of some microRNAs after ubiquinol supplementation might be associated with a reduction in the prothrombotic profile of APS patients [58]. Future studies assessing the effects of various anti-inflammatory and antithrombotic treatments on the expression levels of these ci-miRNAs are required to better clarify the potential role of these ci-miRNAs as therapeutic candidates for thromboprophylaxis in APS and BS.

## CRediT authorship contribution statement

**Alessandra Bettiol:** Writing – original draft, Investigation, Data curation, Conceptualization. **Giacomo Bagni:** Writing – original draft, Visualization, Validation, Methodology, Investigation, Formal analysis, Data curation, Conceptualization. **Francesca Di Patti:** Writing – review & editing, Validation, Methodology, Formal analysis, Data curation. **Elena Lastraioli:** Writing – review & editing, Investigation. **Alice Barinotti:** Writing – review & editing, Resources, Investigation. **Massimo Radin:** Writing – review & editing, Resources, Investigation. **Savino Sciascia:** Writing – review & editing, Investigation, Conceptualization. **Domenico Prisco:** Writing – review & editing, Conceptualization. **Annarosa Arcangeli:** Writing – review & editing, Conceptualization. **Giacomo Emmi:** Writing – review & editing, Supervision, Resources, Conceptualization.

## Independent data access and analysis

ABe, GB and FDP had full access to all the data in the study and take shared responsibility for its integrity and the data analysis.

## Research data

Deidentified research data will be made available upon written request to the corresponding author.

## Sources of funding

This research did not receive any specific grant from funding agencies in the public, commercial, or not-for-profit sectors.

## Declaration of competing interest

The authors declare that they have no known competing financial interests or personal relationships that could have appeared to influence the work reported in this paper.

## Data Availability

Data will be made available on request.
