# Supplementary material for: Epigenetic regulation of thrombo-inflammation in Behçet and antiphospholipid syndrome
Source: J Transl Autoimmun. 2025 May 24;11:100293. doi: 10.1016/j.jtauto.2025.100293 (PMC12169774; doi:10.1016/j.jtauto.2025.100293)
Supplement: Multimedia component 1 [file mmc1.docx]

**SUPPLEMENTARY MATERIAL**

**Figure S1.** Expression levels of miR-206, miR-224-5p and miR-653-5p in BS patients (n=39), APS patients (n=33) and healthy controls (n=30)

*APS: antiphospholipid syndrome; BS: Behçet syndrome; HC: healthy controls; KW: Kruskal Wallis*

*** adjusted p-value<0.01 from head-to-head post-hoc Dunn’s multiple comparison test*

**Figure S2.** Expression levels of miR-206, miR-224-5p and miR-653-5p in BS and APS patients with history of vascular events (n=22 each) and healthy controls (n=30)

*APS: antiphospholipid syndrome; BS: Behçet syndrome; HC: healthy controls; KW: Kruskal Wallis*

** adjusted p-value<0.05 from head-to-head post-hoc Dunn’s multiple comparison test; ** adjusted p-value<0.01 from head-to-head post-hoc Dunn’s multiple comparison test; *** adjusted p-value<0.001 from head-to-head post-hoc Dunn’s multiple comparison test*

**Table S1.** Expression levels of miR-206, miR-224-5p and miR-653-5p in the subgroup of BS and APS patients with vascular involvement, stratified according to therapeutic features at time of enrolment.

|  | **miR-206** | **p-value** | **miR-224-5p** | | **p-value** | **miR-653-5p** | **p-value** |
| --- | --- | --- | --- | --- | --- | --- | --- |
| **BS patients (n=22)** |  |  |  | |  |  |  |
| **Ongoing treatment** |  |  |  | |  |  |  |
| Corticosteroids (Yes: n=10) | No: 3.53 (1.82; 4.14)  Yes: 2.18 (0.87; 3.09) | 0.075 | No: -0.15 (-0.74; 0.94)  Yes: 2.07 (1.19; 2.50) | | 0.041 | No: 3.29 (1.37; 4.20)  Yes: 0.13 (-1.56; 2.1) | 0.048 |
| Traditional DMARDs (Yes: n=13) | No: 2.08 (-0.45; 3.47)  Yes: 3.09 (2.27; 3.72) | 0.271 | No: 0.74 (-0.62; 2.43)  Yes: 1.19 (-0.18; 2.26) | | 0.815 | No: 2.73 (-2.65; 4.18)  Yes: 2.10 (0.41; 3.18) | 0.867 |
| Biological DMARDs (Yes: n=17) | No: 4.18 (3.09; 4.82)  Yes: 2.27 (0.86; 3.36) | 0.021 | No: 2.26 (-0.18; 2.32)  Yes: 0.74 (-0.62; 1.88) | | 0.411 | No: 2.50 (0.83; 3.18)  Yes: 2.10 (-1.44; 3.47) | 0.505 |
| Anticoagulants/antiplatelets (Yes: n=4) | No: 2.34 (0.86; 3.47)  Yes: 3.83 (2.89; 5.06) | 0.106 | No: 1.39 (-0.12; 2.32)  Yes: -0.40 (-0.90; 2.44) | | 0.444 | No: 1.47 (-1.44; 3.47)  Yes: 3.29 (1.80; 4.60) | 0.211 |
| **APS patients (n=22)** |  |  |  | |  |  |  |
| **Ongoing treatment** |  | |  |  |  |  |  |
| HCQ (Yes: n=8) | No: 1.37 (-0.38; 3.10)  Yes: 1.31 (0.27; 2.15) | 0.682 | No: 0.35 (-0.46; 2.95)  Yes: 0.86 (-0.57; 2.52) | | 0.946 | No: 1.90 (-0.03; 5.71)  Yes: 2.69 (0.77; 6.10) | 0.633 |
| Anticoagulants (Yes: n=17) | No: 0.56 (0.36; 1.23)  Yes: 1.45 (-0.38; 3.03) | 0.457 | No: 1.34 (1.31; 3.70)  Yes: 0.07 (-0.72; 2.52) | | 0.170 | No: 4.03 (0.75-7.38)  Yes: 1.75 (0.49; 4.82) | 0.411 |
| Antiplatelets (Yes: n=5) | No: 1.39 (-0.38; 2.57)  Yes: 1.29 (0.56; 2.98) | 0.724 | No: 0,.41 (-0.72; 2.95)  Yes: 0.64 (-0.22; 1.31) | | 0.969 | No: 1.34 (0.49; 4.82)  Yes: 3.14 (2.05; 7.38) | 0.256 |

**Table S2.** Comparison of TGA parameters in 12 cases with APS before initiating anticoagulation, matched by age and sex with 12 BS patients without ongoing anticoagulation.

| TGA parameters | APS before anticoagulation | BS without anticoagulation | *p*-value |
| --- | --- | --- | --- |
| tLag | 9.9±7.5 | 2.5±3.9 | p=0.006 |
| tPeak | 31.2±17 | 22.7±9.7 | p=0.147 |
| Peak | 184.5±123.9 | 239.9±119.5 | p=0.277 |
| AUC | 2256±1445.3 | 2431±1156.6 | p=0.680 |

*APS: antiphospholipid syndrome; BS: Behçet syndrome*
